# A hierarchical clustering approach to identify repeated enrollments in web survey data

**DOI:** 10.1371/journal.pone.0204394

**Published:** 2018-09-25

**Authors:** Elizabeth A. Handorf, Carolyn J. Heckman, Susan Darlow, Michael Slifker, Lee Ritterband

**Affiliations:** 1 Biostatistics and Bioinformatics Facility, Fox Chase Cancer Center, Philadelphia, PA, United States of America; 2 Department of Medical Oncology, Rutgers Cancer Institute of New Jersey, New Brunswick, New Jersey, United States of America; 3 National Comprehensive Cancer Network, Fort Washington, PA, United States of America; 4 Department of Psychiatry and Neurobehavioral Sciences, University of Virginia, Charlottesville, VA, United States of America; University of Michigan, UNITED STATES

## Abstract

**Introduction:**

Online surveys are a valuable tool for social science research, but the perceived anonymity provided by online administration may lead to problematic behaviors from study participants. Particularly, if a study offers incentives, some participants may attempt to enroll multiple times. We propose a method to identify clusters of non-independent enrollments in a web-based study, motivated by an analysis of survey data which tests the effectiveness of an online skin-cancer risk reduction program.

**Methods:**

To identify groups of enrollments, we used a hierarchical clustering algorithm based on the Euclidean distance matrix formed by participant responses to a series of Likert-type eligibility questions. We then systematically identified clusters that are unusual in terms of both size and similarity, by repeatedly simulating datasets from the empirical distribution of responses under the assumption of independent enrollments. By performing the clustering algorithm on the simulated datasets, we determined the distribution of cluster size and similarity under independence, which is then used to identify groups of outliers in the observed data. Next, we assessed 12 other quality indicators, including previously proposed and study-specific measures. We summarized the quality measures by cluster membership, and compared the cluster groupings to those found when using the quality indicators with latent class modeling.

**Results and conclusions:**

When we excluded the clustered enrollments and/or lower-quality latent classes from the analysis of study outcomes, the estimates of the intervention effect were larger. This demonstrates how including repeat or low quality participants can introduce bias into a web-based study. As much as is possible, web-based surveys should be designed to verify participant quality. Our method can be used to verify survey quality and identify problematic groups of enrollments when necessary.

## 1. Introduction

Public health researchers are increasingly using internet-based surveys and interventions in their research. There are many benefits to web-based interventions, including the ability to personalize, to more easily create disseminable public health interventions, and to economically reach a large number of research participants. Additionally, with approximately 99% of U.S. young adults using the internet [1] and the evidence for the efficacy of internet interventions [2], the internet is an appropriate modality with which to reach young adults and to test public health interventions targeted at this population.

An issue unique to web-based surveys, particularly those with monetary incentives, is that participants may enroll multiple times to obtain the incentives. This phenomenon has been previously observed in internet survey research, such as a case study by Konstan et al [3], where through a combination of email addresses, payment records, IP addresses, and other quality indicators, the study team determined that 11% of their sample consisted of repeat responses, including 1 subject who enrolled 65 times.

Generally, any anonymous survey-based research (web-based or otherwise) may be subject to data quality issues beyond typical errors in self-reported data. Participants may not thoughtfully complete the questionnaire, a phenomenon termed “careless responding.” Meade and Craig developed a method to evaluate survey responses to identify participants with low-quality survey data [4]. They suggest quantifying response consistency, the presence of outliers, and response time, and using a latent class model to determine which subjects may have responded carelessly.

In this paper, we develop methods to systematically identify non-independent enrollments (i.e. a single individual enrolled in a study multiple times) when there is no way to confirm subjects’ identities. We provide a novel simulation-based method for identifying such participants via hierarchal clustering methods, and demonstrate how it can be used in the context of web-based surveys. We then compare our findings with other methods used to measure survey quality, and demonstrate how inclusion of clustered participants may change study findings.

## 2. Identification of similar response patterns

### 2.1 Motivating study

This work was motivated by enrollment quality issues that arose during the conduct of a web-based interventional study, designed to change participant behaviors associated with risk of developing skin cancer. In this study, named UV4.me, the team developed the first web-based intervention to modify skin cancer risk and protective behaviors targeted specifically for young adults, which was informed by the Integrative Model for Behavioral Prediction (IM) [5]. The UV4.me intervention was targeted to young adults, personally tailored, and included interactive, multimedia, and goal-setting components. Study methods and interventions have been described previously [6]. Briefly, participants were recruited nationally online by a consumer research company, using their US consumer opinion panel and partnerships with other panels and online communities. Panelists were exposed to brief web banner ads about the study from which they could click to link to the study website. Once at the study website, interested candidates were asked to complete the Brief Skin Cancer Risk Assessment Tool (BRAT) [7], which was scored automatically. Eligible participants were 18–25 years old, had never had skin cancer, and were at moderate to high risk of developing skin cancer based on the BRAT [7]. In a national randomized controlled trial [6], the UV4.me intervention was found to be efficacious in significantly decreasing ultraviolet radiation exposure and increasing skin protection behaviors among young adults at risk of skin cancer. The effects of the intervention have been previously described in detail elsewhere [6, 8]. This project was approved by Fox Chase Cancer Center's IRB, and electronic informed consent was obtained from all research participants.

During enrollment of subjects into the UV4.me study, the research team detected individuals who attempted to enroll in the study multiple times. Some re-enrollments were clear and could be manually removed, as when multiple enrollments used the same name or email address. Later in the study, based on email interactions with study participants, the team noticed a series of suspicious enrollments. These participants appeared to be coming from outside of the US, which would make them ineligible, but this could not be objectively confirmed. The participants used different email addresses and names for the enrollments; however, their responses to the screener questionnaire were unusually consistent. The team was unable to find a single unique identifier that could separate these problematic enrollments from valid participants; IP addresses, for example, were not available. We therefore sought to develop an objective criteria based on multiple factors by which we could exclude repeat enrollments. In our main analysis of intervention efficacy, we used clustering and latent class models in attempt to remove the most problematic enrollments [6]. In this work, we further develop these methods, and propose a novel simulation-based approach to objectively detect unusual clusters of enrollments.

Every subject interested in participating in the UV4.me study filled out the Eligibility Screener (ES) to determine whether they meet the study inclusion criteria. A key portion of the ES was the BRAT, a validated instrument which identifies subjects at high risk of developing skin cancer [7]. The eight Likert-type items on this scale are weighted according to the amount of risk that each characteristic conveys, and are summed to give a single aggregate risk score. In the UV4.me study, participants were eligible if they were at moderate to high risk of skin cancer with a total risk score of 27 or more (out of a possible 89). The items, their scoring, and the proportion of participants in the final eligible sample are given in supplementary S1 Table. Due to the large number of potential response patterns for the BRAT questionnaire, it provided an opportunity to identify unusually similar enrollments. In addition to the BRAT items, we also considered self-reported age. Further, as problematic subjects appeared to be clustered in time (Supplementary S1 Fig), we also considered order of enrollment. Other items in the ES, such as prior skin cancer diagnosis and state of residence in childhood were excluded due to sparseness or lack of variability.

### 2.2 Methods

#### 2.2.1 Hierarchical clustering

**Fig 1:**
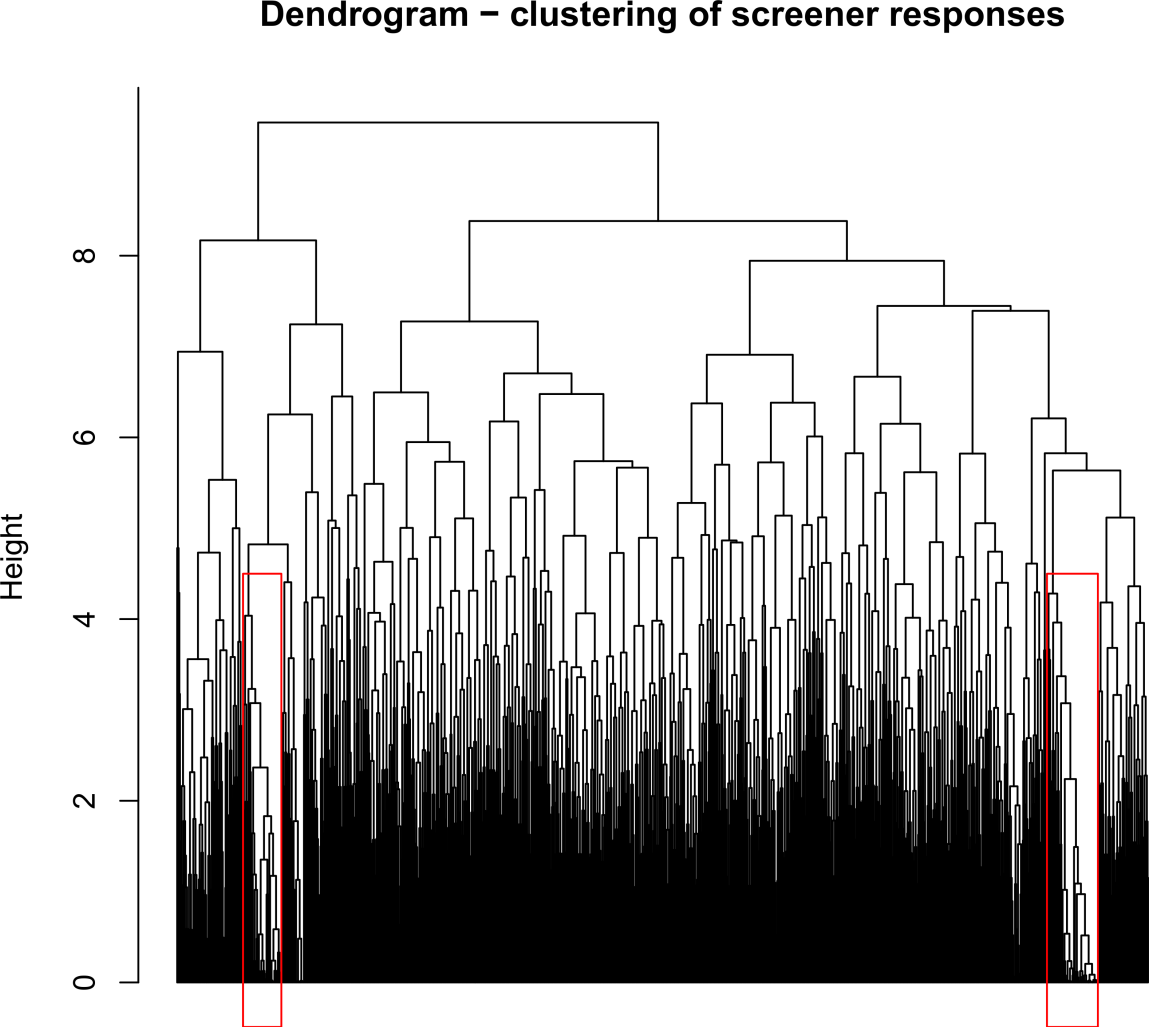
Dendrogram representing clustering of screener responses in participants who went on to complete the baseline questionnaire.

We identified groups of similar enrollments from the 1,234 participants who met all study inclusion criteria by applying hierarchical clustering [9] to their responses on the ES. Hierarchical clustering is a common method for clustering high-dimensional data, which uses an agglomerative (“bottom-up”) approach based on a dissimilarity (e.g. distance) measure between each pair of observations. The algorithm begins with each observation in a different cluster and proceeds iteratively, joining the two most “similar” clusters at each step. Similarity can be defined in several ways, one common choice being complete linkage. Under this definition, the maximum dissimilarity between each pair of observations in the two candidate clusters is calculated. Therefore, the clusters which are joined minimize the maximum within-cluster distance. This value then becomes the “height” at which the two clusters merged, which can then be displayed using a dendrogram (see Fig 1), with height represented on the y-axis. Cluster membership can be defined by choosing a height threshold; any observations that are joined at a smaller value than the chosen height are considered to be in the same cluster. The choice of height therefore defines the number and membership of clusters.

For the purposes of defining dissimilarity, we treated the ordinal Likert-type items as continuous. We constructed a Euclidean distance matrix using the eight fields in the ES, age, and enrollment order after standardizing the items to their corresponding standard deviation. This distance matrix formed the dissimilarity measures used by the hierarchical clustering algorithm. Formally, the distance between any two participants, *i* and *j*, was defined as

$$d_{ij}=\sqrt{\sum_{k=1}^{10}{\left(\frac{x_{jk}-x_{ik}}{\sigma_{k}}\right)}^{2}},$$

where *x*_*ik*_ and *x*_*jk*_ are the value of the *k*^th^ field observed for participants *i* and *j*, respectively, and *σ*_*k*_ is the standard deviation of the *k*^th^ field. We applied the hierarchical clustering algorithm with complete linkage to the resulting distance matrix ***D***, consisting of all pairs of elements *d*_*ij*_.

Cluster size was defined by the number of participants in a cluster at a given height. We measured within-cluster similarity using the average silhouette (sil) width. Sil width is a measure of similarity of an observation to others within its identified cluster, compared to those in the closest other cluster. For a given observation *i*, sil width is defined as

$$s(i)=\frac{b(i)-a(i)}{max[a(i),b(i)]},$$

where *a(i)* is the average distance between observation *i* and the other observations in its own cluster, and *b(i)* is the average distance between observation *i* and all other observations in the closest other cluster [10]. Average Sil Width (*ASW*) of cluster *k* is the average of *s(i)* for all observations within that cluster. Larger values of *ASW* indicate better fit, and negative values may indicate that that the number of clusters (here defined by the specified height) is too small or too large.

#### 2.2.2 Simulating cluster characteristics under independence

Testing for evidence of clustering is challenging, and is often done empirically. Suzuki and Shimodaira [11] proposed a bootstrap based procedure for testing stability of clusters in genetic analyses, for example, in DNA microarrays. Unlike a typical bootstrap procedure, instead of resampling the observations (participants) the variables which are used to define distances are resampled instead, and the clustering algorithm is re-calculated with the updated distance matrix. In typical DNA experiments the number of variables is large, but in our application we only have 10 variables on which the clusters are defined, so we cannot reliably use this type of bootstrap resampling.

We therefore propose a simulation based approach, where we generate independent observations based on the empirical distribution of the variables of interest. The hierarchical clustering algorithm is then applied to the simulated dataset, and we summarize resulting clusters using relevant measures of size and similarity. This process is then repeated many times. As the simulated cluster is based on independent data, we can determine the distribution of its characteristics when no clustering is present, and determine if the observed dataset deviates substantially from the expected distribution under independence. Our approach is comparable to that described by Hennig et al. [12] for k-medoid clustering, a “top-down” clustering method where all observations are fit into a number of pre-specified clusters, *k*. However, their goal was to identify stable clusters encompassing the whole dataset, while our objective was to identify large, unusually similar groups of responses. Therefore, we considered both cluster size and within-cluster similarity, as quantified by *ASW*. Key to this approach is the simulation of the “null” distribution, from non-clustered, independent observations (Steps 2–3). For Likert-type items, we recommend using the GenOrd R package, or another comparable tool. This program allows the user to specify the observed marginal proportions of each ordinal category, and the observed correlation matrix [13]. It then simulates data from the multivariate normal distribution, and these continuous values are categorized based on quantiles of the normal distribution such that the simulated samples have the same proportions as the observed data. This program also applies a correction to the correlation matrix used to generate the multivariate normal data, so that when the simulated values are categorized as ordinal, the correlation matrix reflects that of the original ordinal data.

Because of the restriction imposed by requiring total score of ≥27 points, we could not directly use the marginal response rates to simulate observations based on the final sample of eligible individuals, as by chance, some simulated responses would not have been eligible for inclusion. Instead, we drew from the full distribution of all responses to the ES from all individuals in the acceptable age range without a personal history of cancer, and then applied the score restriction. Some participants who met the score restriction did not go on to complete the Baseline Questionnaire (BQ). We modeled the probability of completing the BQ in this population based on age and answers to the BRAT questionnaire using logistic regression. For each simulated sample, we estimated the probability that they would complete the BQ, and then used this estimate to randomly assign them as completing/not completing the BQ. We used rejection sampling (based on simulated scores and BQ completion indicators) until we obtained a sample of 1,234 simulated observations with valid scores. Enrollment order was then permuted, assuming that enrollment characteristics would be stable over time under independence. This procedure was repeated 1,000 times, and performed the hierarchical clustering algorithm on each of the simulated datasets.

To identify repeat enrollments, we wished to find clusters with unusual within-cluster similarity and large size. Small clusters tend to have wide variability in similarity measures (ranging from low to high similarity), while large clusters tend to have smaller within-cluster similarity. We therefore proposed the following measure Size and Sil Width (*SSW*) for each cluster *c* (out of all clusters *C*).

$${SSW}_{c}=\frac{N_{c}}{\max_{j\in C}\left(N_{j}\right)}+\frac{{ASW}_{c}}{\max_{j\in C}\left({ASW}_{j}\right)},$$

where *N* is the number of samples in the cluster, and *ASW* is the average sil width. As these measures are on different scales, we standardize them by dividing by the maximum observed values for all clusters, so that both components are equally important. This measure is highest for large, similar clusters. We then calculate this statistic for the simulated samples as

$${SSW}_{c}^{*}=\frac{N_{c}^{*}}{\max_{j\in C}\left(N_{j}\right)}+\frac{{ASW}_{c}^{*}}{\max_{j\in C}\left({ASW}_{j}\right)},$$

where N* and ASW* are the sizes and average sil widths of the clustered samples. We normalize them to the maximum sizes and sil widths of the original samples to maintain the same scale. We then compare the *SSWs* for the original dataset to distribution of $\max({SSW}_{c}^{*})$, the maximum *SSW*s observed in each of the simulated samples.

#### 2.2.3 Summary of algorithm

Below, we summarize the steps used to identify unusual clusters. R code for implementation of this procedure is available in the supplementary materials (S1 Code).

1. Perform hierarchical clustering on original dataset (***X)*** where the columns are the *K* variables used to define clusters, and the rows are the *N* samples.
  - Calculate the Euclidean distance matrix *D*, were the elements of *D* are defined by $d_{ij}=\sqrt{\sum_{k=1}^{K}{\left(\frac{x_{jk}-x_{ik}}{\sigma_{k}}\right)}^{2}}$ (Euclidean distance between every pair of individuals).
  - Run a hierarchical clustering analysis on *D*.
  - Apply several height thresholds to partition the samples into clusters.
  - Choose a height threshold which results in good fit (e.g. as measured by *ASW*).
2. Let *V*_***i***_ be the ith variable (column) in ***X*.** Estimate the multivariable distribution ${\hat{F}}_{N}(V)$ assuming independence between samples (the “null” distribution).
3. Draw *N* independent samples, such that V* i.i.d. ${\hat{F}}_{N}(V)$, to create a simulated dataset X*.
4. Repeat the simulation procedure (step 3) *M* times.
5. Apply the hierarchical clustering procedure to the *M* simulated datasets (X*).
  - Repeat step 1a-b for each X*.
  - Using the height threshold chosen in 1d, partition the *N* rows into clusters.
6. Compare the observed clusters found in step 1 to the simulated clusters from step 5.
  - Quantify size and within-cluster similarity (*SSW*) of each cluster *c* found in step 1 (based on ***X***).
  - For each of the *M* simulated clusters, find $\max({SSW}_{c}^{*})$.
  - For each cluster *c* from the observed data, calculate $Pr({SSW}_{c}>\max({SSW}_{c}^{*}))$, the proportion of the times the observed *SSW* is greater than the maximum *SSW*s from each of the *M* simulations.

Note that steps 2–4 are similar to those proposed by Hennig et al. [12] to assess the properties of k-medoid clustering using a simulated “null” distribution. Our method mainly differs in steps 1, 5, and 6, as we need to evaluate the properties of each observed cluster, not overall fit.

### 2.3 Results

Applying the hierarchical clustering approach described in Section 2.2.1, we created a dendrogram to illustrate the structure of the study data (Fig 1). A figure showing the structure of Euclidean pairwise distances is available in the supplementary files (S1 Fig).

Visual inspection of the dendrogram shows that there are two clusters which seem to have a large degree of similarity compared to the remainder of the population. The clusters are unusual in that they have especially low dissimilarity and large size. As cluster definition depended on height, we explored several thresholds: 4.5, 5.0, and 5.5. Based on the observed *ASWs*, we chose a height of 4.5, as *ASWs* were more often negative for the larger heights. We did not explore smaller height thresholds as we wished to split the dataset into the fewest possible number of clusters. This resulted in a set of 80 clusters, ranging in size from 1 to 65 participants per cluster, with *ASWs* ranging from -0.042 to 0.441.

**Fig 2:**
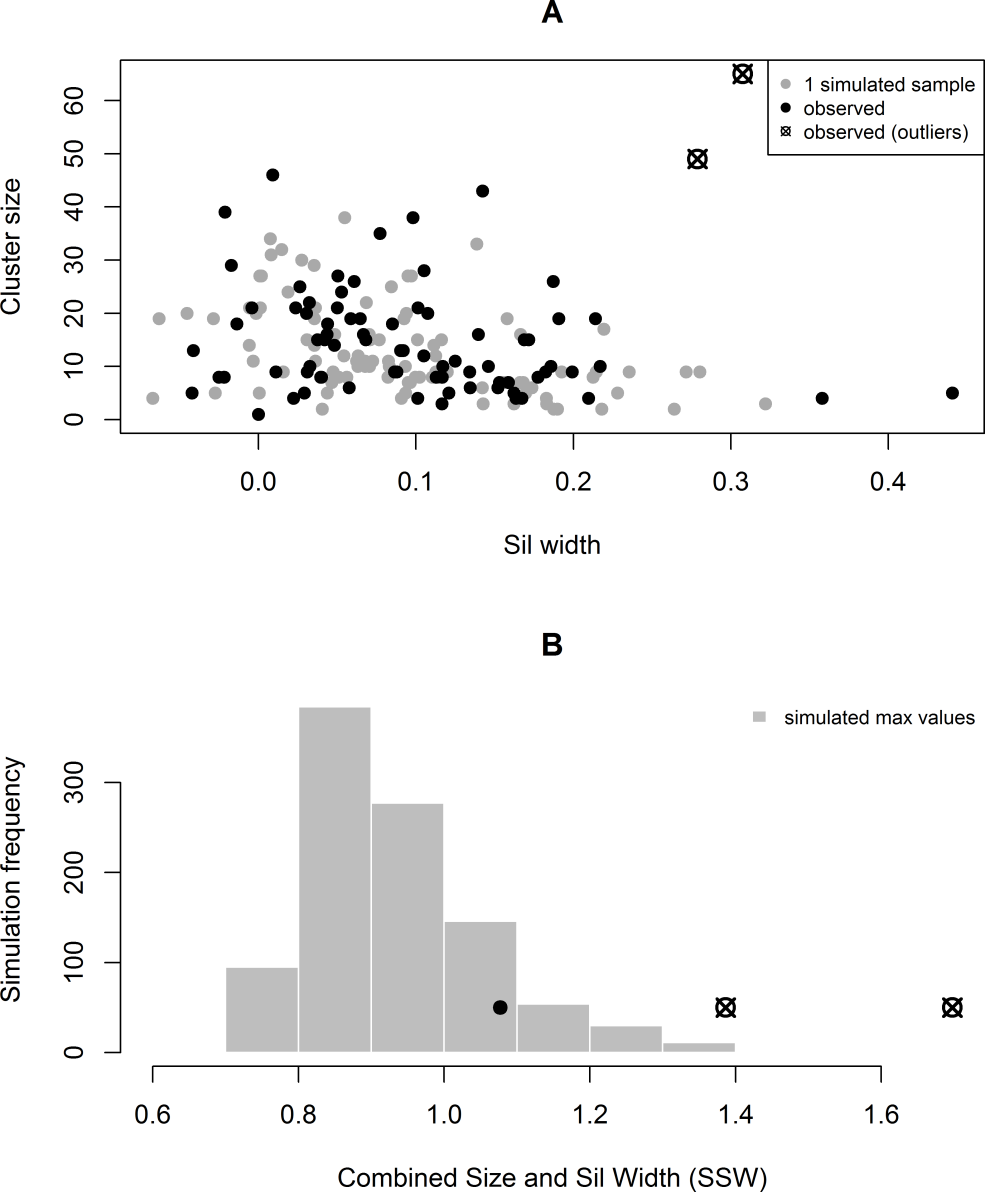
(A) shows the distribution of the observed cluster sizes and sil widths (black) compared to the distribution of size and sil widths from one simulated sample. (B) shows the distribution of the maximum *SSWs* from each of 1,000 simulations, compared to the top three observed *SSWs* in the true data. The two outliers of interest are represented by an X through a circle in both plots.

The distribution of *N* versus *ASW* and *SSW* from the original data and a single simulated dataset are shown in Fig 2A. Gray points are from the simulated dataset and black points are from the original dataset. We note that most of the clusters in the observed data have similar characteristics to those of the simulated clusters; however, this method detected two outlying clusters, consisting of 65 and 49 participants each. After calculating the maximum observed *SSWs* from 1,000 simulated datasets, we found that these clusters had *SSWs* greater than 100% and 99.6% of the simulated values of $\max({SSW}_{c}^{*})$, respectively. The cluster with the next largest *SSW* only had values greater than 88.0% of the simulated values. (See Fig 2B) Although this method does not give a true p-value, it provides substantial evidence that these clusters would be unlikely to occur by chance if the data were truly independently distributed.

#### 2.3.1 Verification of algorithm

Next, we evaluated our method of identifying non-independent enrollments in two ways. First, we re-ran the algorithm including participants who were previously dropped for known issues (N = 257), including one participant who re-enrolled 53 times (detected by similar nonsense registration names, e.g. “asdf”). The clustering method identified an additional cluster (with 91 total enrollments), including 44 of the 53 enrollments from the known repeater (83%).

Second, we generated synthetic datasets where we introduced a true cluster (with correlated observations), and used our method to see if this known cluster could be identified. By repeating this many times, we evaluated the method’s false positive and false negative rates under different data-generating mechanisms. Due to the computational complexity of this procedure, we explored a limited number of scenarios, and used 100 synthetic datasets per scenario. Most observations in each synthetic dataset were generated independently, using the method described in Section 2.2.2. We also generated a much smaller clustered sample where the correlation matrix (***∑***) consisted of a series of blocks. The blocks along the diagonal, ***S***, defined the correlation of items within an individual. We then defined the off-diagonal blocks to be *r****S***, where 0 ≤ *r* ≤1, which induced a correlation between individuals. R code and a supporting datafile are available in Supplementary files S2 Code and S1 File.

In the synthetic datasets, we specified the number of samples, vector of means and correlation matrices to reflect approximately what was present in the true dataset. We varied *r* to determine how the algorithm performs under varying degrees of correlation. For our base case, we used *r* = 0.8, anticipating that the clustered samples would be highly correlated (i.e. a cluster of repeated enrollments from one individual). We specified that if $Pr({SSW}_{c}>\max({SSW}_{c}^{*}))>0.95$, a cluster was considered to be unusual. The mean number of non-clustered individuals was 1296 and the mean number of individuals in the cluster was 59. The algorithm identified clustering in 74% of the synthetic datasets. When clustering was detected, 88.4% of the clustered samples were identified as unusual by the simulation-based algorithm, leading to an overall sensitivity of 65.4%. Specificity was excellent, with only 0.5% of non-clustered samples falsely identified as belonging to an unusual cluster (specificity = 99.5%). When we reduced the correlation to r = 0.6, sensitivity was 59.7% and specificity was 99.5%. Finally, we tested whether we could improve sensitivity by lowering the value of $Pr({SSW}_{c}>\max({SSW}_{c}^{*}))$ to 0.8; which resulted in increased sensitivity of 70.7% with only a small decrease in specificity (99.4%). In our application, we considered high specificity to be critical, as we wanted to limit the number of true enrollments excluded from the analysis. This motivated our use of the 0.95 threshold; however, these results indicate that lower thresholds could be considered to improve sensitivity.

## 3. Comparison with survey quality measures

### 3.1 Methods

Use of self-reported data is the standard in psychology and behavioral science. However, in addition to the known issues of using self-reported data such as participant memory and study demand characteristics [14], survey participants may not complete questionnaires thoughtfully and carefully, which has been described as “careless responding”. [4] Meade and Craig proposed several metrics which, in combination, can be used to identify careless respondents. For our study, we performed a similar type of analysis to that proposed by Meade and Craig, which we adapted based on measures available for our study. We identified 12 relevant quality measures, which are described in detail in the supplementary files (S2 Table). We adapted five relevant metrics proposed by Meade and Craig, including time to complete the survey, three measures of response consistency/similarity, and patterned responding (repeated identical responses on the same page). We also incorporated seven other indicators of potential problems that were collected by the study team during enrollment and follow-up, including discrepancies between self-reported characteristics (e.g. skin color or gender) at different parts of the questionnaires and non-US phone numbers. Note that the variables used in the clustering procedure above were from the Eligibility Screener (ES), while the quality measures relied on data from the Baseline Questionnaire (BQ), and other measures collected during the enrollment/study process. Therefore, the quality variables were measured separately from variables used to identify clustering.

We performed latent class modeling based on the quality variables to identify underlying groups. Continuous measures were categorized into deciles, as some of the measures were highly skewed, and for ease of fitting a latent class model with mixed variable types. We fit models with two to four classes, and selected the three class model as it minimized the Bayesian Information Criterion (BIC). We then determined predicted class membership for each observation [15]. We also tried including an indicator for membership in one of the clusters as an additional variable in the latent class model; however, these models had worse overall fit as measured by the BIC. See supplementary materials (S) for R code.

### 3.2 Results

The relationship between latent class membership and cluster membership is shown in Table 1. Interestingly, individuals from the two clusters were mainly fit in two separate classes. As the final latent class model did not include an indicator for cluster membership, or depend on any of the variables from the ES, this provides further evidence that these groups of unusual registrations were non-independent. Table 2 describes the quality measures, separately by cluster membership and by latent class membership.

**Table 1:** Cluster membership vs. latent class membership.

Cluster Membership	Latent Class Membership			
	Class 1	Class 2	Class 3	Total
Other*	610	406	104	1120
Cluster #1	7	55	3	65
Cluster #2	2	4	43	49
Total	619	465	150	1234

*“Other” denotes membership in one of the clusters not identified as usually large/similar

**Table 2:** Quality variables by cluster membership and predicted latent class.

Quality variables	Full sample	Cluster Membership			Latent Class Membership		
		Other*	1	2	1	2	3
1. Minutes to complete questionnaire (median)	21	21	14	935	20	15	935
2. Correlation of synonyms (mean) *within-person correlation of items with strongest overall correlation*	0.4	0.39	0.58	0.33	0.28	0.59	0.33
3. Even-odd item correlation (mean) *Correlation of even/odd items on unidimensional scales*	0.62	0.61	0.74	0.66	0.60	0.64	0.62
4. Distance from average (mean) *Euclidean distance between participant’s responses and population average responses*	15.64	15.82	12.85	15.33	17.40	13.45	15.18
5. Runs of identical responses (mean) *Average over pages with many items*	0.43	0.44	0.47	0.27	0.43	0.49	0.27
6. Inconsistent state/climate selection	15.0%	12.9%	16.9%	61.2%	6.8%	13.8%	52.7%
7. Non-US phone	16.0%	15.3%	13.9%	36.7%	13.1%	8.4%	52.0%
8. Wrong phone number	13.0%	13.8%	9.2%	2.0%	8.9%	22.4%	1.3%
9. Obviously fake name	6.8%	6.8%	12.3%	0.0%	0.5%	16.3%	3.3%
10. Nonsensical feedback *e*.*g*. *“dsadasdasd”*	2.8%	2.0%	20.0%	0.0%	0.3%	7.1%	0.0%
11. Discrepancies within questionnaire	4.3%	3.8%	16.9%	0.0%	2.3%	7.7%	2.0%
12. Other	9.3%	9.7%	6.2%	4.1%	7.4%	12.5%	7.3%

Note: Shading emphasizes unusual behavior patterns as measured by the quality indicators

*“Other” denotes membership in one of the clusters not identified as usually large/similar

From the observed values of the quality measures by cluster membership, we see that cluster 1 exhibits higher than average similarity (as quantified by items 2–4), with high correlation of synonyms (items with highest correlation across the whole sample), high correlation of even/odd numbered items in unidimensional scales, and overall responses very close to the mean response (as measured by Euclidean distance). They also completed the questionnaire in shorter times, had high rates of giving fake names, providing nonsensical feedback, and giving inconsistent answers about their characteristics (e.g. skin color or gender). Members of cluster 2 had different patterns of behavior. Their median completion time was very high (indicating that they left their sessions open for a long time before submitting), and their responses had low correlation of synonyms, short runs of identical responses, high rates of inconsistencies between state and climate region of the US, and high rates of giving a non-US phone number.

There was substantial overlap between class and cluster membership, and similarities in overall patterns of behavior. The majority of subjects in cluster 1 (55/65, or 85%) were grouped in latent class 2, although they made up only a small proportion of this class (with 465 total subjects). Likewise, the majority of subjects in cluster 2 were fitted into latent class 3 (43/49, or 88%). Cluster 2 made up a larger proportion of latent class 3, although they were still a minority of the total cluster membership (142 total subjects). It is interesting to see that many subjects who were not identified as members of one of the clusters had similar behavior patterns as the clustered subjects, as shown by the quality indicators in Table 2.

## 4. Implications for study findings

The goal of the UV4.me intervention was to increase beneficial behaviors (skin protection) and decrease detrimental behaviors (ultraviolet radiation [UV] exposure) related to skin cancer. As described in prior work [6], we used linear regression models to identify the effect of the intervention versus the control condition (assessment only) at the time of the final questionnaire (12-weeks post enrollment). We accounted for within-subject correlation across the measurement times using robust sandwich variance estimates with Generalized Estimating Equations (GEE). The linear regression model included main effects for treatment and time (both categorical), and interactions between treatment and time. The model used an auto-regressive working correlation structure with clusters defined by patients [16]. See supplementary file S3 Code for R code to run these models.

Table 3 shows the results in the full sample (N = 1,234), in the participants who were not members of one of the unusual clusters (N = 1,120), and in participants with predicted membership in the best quality latent class (Class 1, N = 622).

**Table 3:** Intervention effects on primary outcomes at 12 weeks, by cluster and latent class membership.

UV exposure outcome	Effect	SE	95% CI		P-val
All participants	-0.19	0.054	-0.30	-0.09	0.0003
Only non-clustered participants	-0.24	0.058	-0.36	-0.13	<0.0001
Only members of latent class 1	-0.31	0.081	-0.47	-0.15	0.0001
**Skin protection outcome**	**Effect**	**SE**	**95% CI**		**P-val**
All participants	0.31	0.081	0.15	0.47	0.0001
Only non-clustered participants	0.32	0.085	0.16	0.49	0.0001
Only members of latent class 1	0.58	0.116	0.35	0.81	<0.0001

Although the intervention had a statistically significant effect regardless of the exclusions, the strength of the effect varied. The effect on exposure was substantively stronger when we removed the 114 participants clustered based on their screener responses, although the effect on protection outcomes was largely unchanged. For both outcomes, the intervention effect was much larger in the subgroup of participants in latent class 1 (best quality class); however, using only this subgroup excludes almost half of otherwise eligible participants, leading to concerns about generalizability.

## 5. Conclusions

In this work, we proposed a method to identify groups of non-independent study participants, as might result from a single individual repeatedly enrolling in a web-based study. Unlike other procedures based on clustering algorithms [10, 11], our method is designed to separate non-independent clusters from a mixed population of clustered and non-clustered data, and can be used when the number of variables which defines distance between pairs of observations is small.

One limitation of the procedure we used to identify clusters is that distribution of the test statistic generated via simulation assumed independence. This study recruited participants using web advertisements and participant referrals, which could lead to some dependence between subjects. Nevertheless, the clustering procedure successfully revealed unusual groups of participants with characteristics not easily explained by modest sources of within-group correlations. Another limitation of this method is that it can only identify large clusters of non-independent enrollments; however, it is most important to be able to detect large groups of repeated enrollments as these are most likely to cause substantial changes in study results. Finally, our method of cluster identification demonstrated excellent specificity; however, further development is needed to improve sensitivity. Future work should determine whether an optimal threshold for identifying unusual clusters can be found, or if modifying the specification of the hierarchical clustering algorithm (i.e. distance metrics and linkage) can improve sensitivity while maintaining specificity.

We compared our results to an adapted version of the method used to identify careless respondents proposed by Meade and Craig [4]. In our application, that approach lacked specificity needed to identify careless responders; however, we were not able to directly replicate all their measures. Although we followed the principles outlined in their method, we could not directly use some of the proposed measures, such as proposed “bogus items” (questions with answers which were obviously wrong). We instead included our own study specific quality measures. Further, due to the discrete nature of some of our measures, we fit latent class models instead of using latent profile analysis. This may have led to some of the differences between our results and those described in the example given in the original paper. Only about half of our samples were fit into the “best quality” cluster, whereas in Meade and Craig’s application, almost 90% of samples were in a single class.

We recommend that all web-based studies are designed with the ability to verify data quality in mind. Kramer and colleagues provide a set of recommendations to decrease enrollment of otherwise ineligible participants [17]. Ideally, sufficient information would be gathered to identify repeat enrollments automatically during the course of the study, not manually or in a post-hoc analysis. As much as is practical, researchers should use metrics that uniquely identify individuals. In the current study, we were not able to obtain information on IP addresses or other unique identifiers. However, such methods are not infallible, as a determined individual can use software to circumvent such identifiers (for example, by varying their IP address) [18]. Further, for socially sensitive topics, researchers may opt not to collect potentially identifying information for the privacy of participants and to minimize the risk of accidental disclosure of participant data. Our clustering method should be used in conjunction with other best practices to look for evidence of repeat enrollments, and potentially exclude problematic subjects.

## Supporting information

S1 Table Table of screening questions from the BRAT scale. (DOCX)

S2 Table Quality measures in detail. (XLSX)

S3 Table Data dictionary (for S1 and S2 Datasets). (DOCX)

S1 Fig Euclidean distance matrix based on screener responses. Red indicates small pairwise distances, blue indicates large distances.
(TIFF)

S1 Dataset Data used in clustering analysis. (CSV)

S2 Dataset Data used in quality and outcome evaluation. (CSV)

S1 Code R code to implement analysis in section 2 (cluster identification). (R)

S2 Code R code to implement sensitivity and specificity analysis in section 2.3.1. (R)

S3 Code R code to implement analysis in sections 3–4 (response quality, effect on outcomes). (R)

S1 File Supporting RData file for S2 Code. (ZIP)
