# Supplementary material for: A hierarchical clustering approach to identify repeated enrollments in web survey data
Source: PLoS One. 2018 Sep 25;13(9):e0204394. doi: 10.1371/journal.pone.0204394 (PMC6155511; doi:10.1371/journal.pone.0204394)
Supplement: S1 Table — (DOCX) [file pone.0204394.s001.docx]

| **Item** | **Data type** | Responses: percent of eligible participants  ( BRAT scale scoring) | | | | | |
| --- | --- | --- | --- | --- | --- | --- | --- |
|  |  | 1 | 2 | 3 | 4 | 5 | 6 |
| How many moles do you have on your body that are larger than a pencil eraser? (1=none, 5=10 or more) | Likert (1-5) | 26.7 (9) | 40.7 (5) | 17.3 (10) | 13.3 (20) | 2.1 (30) | - |
| How many freckles did you have before age 18? (1=none, 3=many) | Likert (1-3) | 14.2 (0) | 51.7 (2) | 34.1 (4) | - | - | - |
| What was the climate of the state or country (Non-United States) where you spent most of your time during childhood? (1=northern, 3=tropical) | Likert (1-3) | 52.3 (0) | 41.2 (5) | 6.5 (10) | - | - | - |
| How many times have you had a very bad sunburn that blistered? (1=none, 5=10 or more) | Likert (1-5) | 10.2 (0) | 32.3 (1) | 31.8 (2) | 18.3 (3) | 7.5 (4) | - |
| What is the color of your untanned skin? (1=very fair, 6=dark brown/black) | Likert (1-6) | 22.0 (20) | 53.7 (18) | 12.2 (16) | 10.4 (4) | 1.6 (2) | <0.1 (0) |
| What was your natural hair color as a teen? (1=red, 5=black) | Likert (1-5) | 4.6 (4) | 27.9 (3) | 35.7 (2) | 25.9 (1) | 5.8 (0) | - |
| How dark of a tan do you get after one week in the summer sun? (1=none, 5=dark) | Likert (1-5) | 10.3 (3) | 32.7 (2) | 47.9 (1) | 9.1 (0) | <0.1 (0) | - |
| Do you burn easily in the sun? (1=Yes, 2=No) | Binary | 69.5 (3) | 30.5 (0) | - | - | - | - |
