# Supplementary material for: A hierarchical clustering approach to identify repeated enrollments in web survey data
Source: PLoS One. 2018 Sep 25;13(9):e0204394. doi: 10.1371/journal.pone.0204394 (PMC6155511; doi:10.1371/journal.pone.0204394)
Supplement: S3 Table — (DOCX) [file pone.0204394.s003.docx]

| **S3 Table: Quality Measures**  Items 1-5 Adapted from Meade and Craig, items 6-12 are study-specific | | |
| --- | --- | --- |
|  | **Measure** | **Description** |
| Baseline questionnaire | 1. Minutes to complete questionnaire | Continuous measure of minutes to complete baseline questionnaire. |
| Baseline questionnaire | 2. Correlation of "synonyms" | Within-respondent correlation of high-correlation items (across the entire sample). Consistency indicator. E.g., I tan to fit in with the crowd, I tan to be like my friends |
| Baseline questionnaire | 3. Consistency of even and odd items | Within-respondent correlation of even and odd items within subscales. |
| Baseline questionnaire | 4. Distance from mean response | Mahalanobis distance between an individual’s responses and the population average to identify outliers |
| Baseline questionnaire | 5. Runs of identical responses | Counts of the number of items in a row with identical responses. Averaged across pages with 4-11 questions on a single page, weighted by the number of questions. Measure of patterned responding. |
| Baseline questionnaire | 6. State and climate inconsistent | Binary indicator for inconsistency between self-reported state and climate (climate definition depends on state). E.g., State = Florida, Climate= Northern |
| Within or across questionnaires | 7. Within study discrepancies | Binary indicator for any discrepancy between self-reported hair color, skin color, sex, age, number of moles, tendency to tan, and tendency to burn (asked at different parts of the study). |
| Eligibility screener | 8. Non US phone number | Binary indicator for registration with a phone number that does not conform to US standards. |
| Eligibility screener | 9. Wrong phone number | Binary indicator. E.g., Call did not go through. Voicemail used a different name. Person who answered the phone did not recognize “participant’s” name. |
| Eligibility screener | 10. Fake name | Binary indictor for obviously fake name - E.g., “No Poop” |
| Follow up questionnaire | 11. Nonsensical feedback | Binary indicator for nonsensical feedback about the program. E.g., Least helpful part of the program – “dsadasdasd”, “great” |
| Various | 12. Other potential quality indicator | Binary indicator for any of these rare (<2% of the sample) instances of unusual responses or behaviors, including 1) Invalid email address, 2) Mismatch between registration name and name included in email address, 3) Participant made second registration attempt after first registration, 4) Possible duplicate (e.g., similar and unusual name or email address), and other similar indicators |
