# Supplementary figures and images for: A hierarchical clustering approach to identify repeated enrollments in web survey data
Source: PLoS One. 2018 Sep 25;13(9):e0204394. doi: 10.1371/journal.pone.0204394 (PMC6155511; doi:10.1371/journal.pone.0204394)

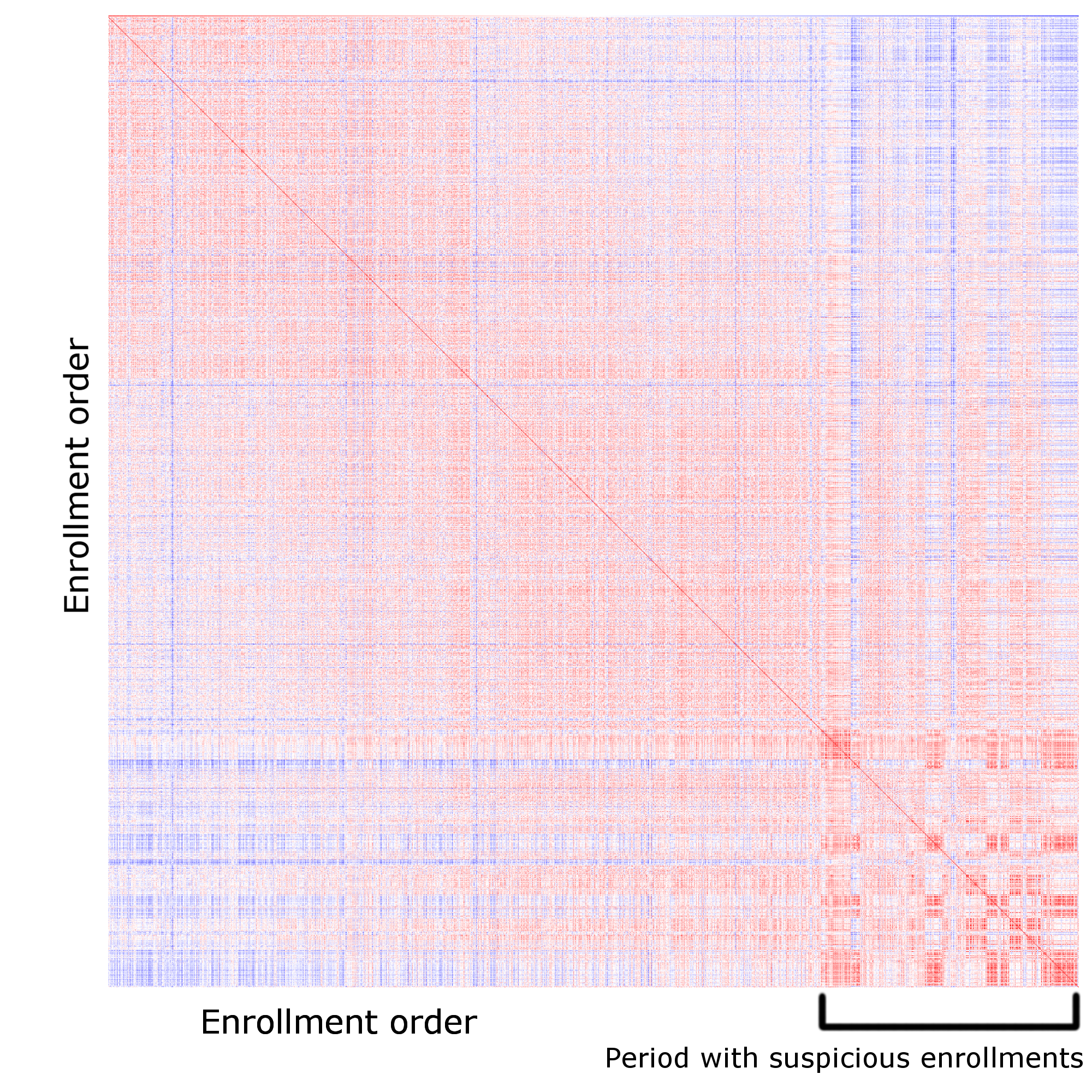

Supplement: S1 Fig — Red indicates small pairwise distances, blue indicates large distances. (TIFF) [file pone.0204394.s004.tiff]
